# Sports poverty alleviation: concept and model innovation for the development of Chinese sports towns in the new Era

**DOI:** 10.3389/fspor.2024.1423767

**Published:** 2024-11-13

**Authors:** Sisi Liu, Wei Qi

**Affiliations:** ^1^ School of Journalism and Communication, Beijing Sport University, Beijing, China; ^2^ School of Humanities and Art, Shenyang University of Technongy, Shenyang, China

**Keywords:** poverty alleviation through sports, sports characteristic town, development momentum, model innovation, rural

## Abstract

In recent years, sports-themed towns have emerged as a key component in China's rural revitalization and poverty alleviation strategy, integrating sports, culture, tourism, and health industries to drive regional economic and social development. However, the literature often overlooks their long-term impact on social, cultural, and community development. Research aims to explore the concept of “sports poverty alleviation” and propose a new development model for sports towns in China, focusing on economic growth as well as social and cultural enhancement, public health, community integration. The study employs mixed methods, including documentary analysis and empirical research, to evaluate the effectiveness of various sports town models in poverty alleviation. Findings reveal that while these towns successfully boost short-term economic growth, especially through tourism and events, their sustainability is challenged by homogenization and a lack of competitiveness. Many towns fail to capitalize on local cultural and natural resources, and while they contribute to immediate economic benefits, they often lack long-term employment and integration strategies for local communities. Successful cases like the Mashan model demonstrate how integrating local resources and cultural identity with sports industries can lead to sustainable development and poverty alleviation. The research concludes that a more holistic approach to the development of sports towns in China is needed. Economic benefits should be complemented by a focus on social, cultural, and community development to ensure long-term success. The proposed innovative development models offer practical solutions to enhance the uniqueness and sustainability of sports towns, contributing to China's rural revitalization and poverty alleviation efforts.

## 1 Introduction

Characteristic towns are new industrial layout forms that emerge at a certain stage of modern economic development, characterized by distinct industrial features, integrated urban and cultural functions, and intensive and efficient space utilization. Sports characteristic towns, as comprehensive development entities integrating numerous industries, resources, and cultures, serve as an effective approach to improving the rural ecological environment, promoting rural industrial upgrading, and enhancing the quality of life of villagers. In 2016, the Jiangsu Provincial Sports Bureau first used the concept of “Sports and Health Characteristic Towns” in its Notice on Launching the Construction of Sports and Health Characteristic Towns. In May 2017, the General Administration of Sports of China issued the Notice on Promoting the Construction of Sports and Leisure Characteristic Towns, which was the first time at the national level that the concept of “Sports and Leisure Characteristic Towns” (referred to as sports characteristic towns) was explicitly proposed and defined as a space area integrating various functions, such as sports and leisure, culture, health, tourism, elderly care, and education and training. It is a platform for the development of national fitness and a base for the sports industry, aimed at promoting the construction of a moderately prosperous society in all respects, supporting new urbanization and the Healthy China Initiative, and facilitating poverty alleviation. In 2018, the General Administration of Sport of China and the State Council Poverty Alleviation Office jointly issued Implementation Opinions on the Sports Poverty Alleviation Project, proposing to give full play to the sports industry's unique advantages in the fight against poverty. The sports characteristic towns are an important measure for sports poverty alleviation, which provides new concepts and impetus for the development of sports characteristic towns on the one hand, and innovative paths for rural revitalization and precision poverty alleviation on the other.

The release of the aforementioned documents indicates the Chinese government's emphasis on the construction of “sports towns”. This also reflects the significant role of sports towns in the future layout and development of China's sports industry. Encouraged by national industrial policies, commercial capital, and local development needs, the construction of characteristic towns in China surged from 2017 to 2021, leading to the emergence of sports towns specializing in football, marathons, and martial arts across the country. Chinese scholars have conducted in-depth research on the relationship between rural revitalization and sports characteristic towns, functional positioning and types of sports characteristic towns, their development models, and optimization paths, yielding partial results. For instance, within the context of the rural revitalization strategy, the construction of sports characteristic towns has been recognized as a concrete measure to implement the strategy, with significant implications for the transformation and upgrading of the township economy and the integration of urban and rural industries (1, 2). The construction of sports characteristic towns serves as a link between rural and urban areas, creating a diverse industrial cluster centered around sports, reconstructing the regional spatial development layout, and promoting the development of remote suburbs and mountainous areas, thereby narrowing the urban-rural development gap (3–5). The development of sports characteristic towns should have a clear industrial orientation, integrating ecological environment, modern technology, tourism landscapes, and distinctive cultures, while also providing community functions such as participation in exercise and leisure for residents (6, 7). Based on this, sports characteristic towns can be categorized into “event-oriented”, “leisure-oriented”, “health-oriented”, and “industry-oriented” types (8). The development of sports characteristic towns has broadened the overall platform of the sports industry, accelerating the transformation and upgrading of the sports industry structure to some extent. It plays a crucial role in expanding the space for national fitness programs, integrating related resources within the field, improving sports service quality, and innovating the dissemination of the concept of sports and leisure (9–11). In accordance with the national requirements for the planning and construction of characteristic sports towns, an analysis of the current issues and strategies in the development of sports towns can be conducted from seven aspects: government management, resource development, event organization, brand image, infrastructure construction, talent cultivation, and ecological environment. This analysis aimed to propose a development model and optimization path for promoting and optimizing the development of sports characteristic towns in China (12–15). The above scholars’ research either discusses the promotion role of the construction of sports characteristic towns on the development of rural industries from the perspective of economics, or analyzes the functions and development models of sports characteristic towns from the perspective of management, or expounds the influence of sports characteristic towns on sports concepts and sports brand culture from the perspective of communication, etc. The research fields are relatively single and lack systematic research. As the construction of sports towns in China started late and is greatly influenced by national policies, there are still certain uncertainties in the role orientation, function and value of sports towns under the background of rural revitalization strategy. Urban-rural integration, industrial integration and effect have not been fully reflected, so it is necessary to fully consider China's reality and take national policies as the guide to find new development impetus and vital development models for sports towns in China.

## 2 Literature review

Existing literature on the relationship between sports-themed towns and targeted poverty alleviation primarily focuses on their economic functions and industrial structures. It is generally believed that sports towns can promote regional economic development and urban-rural integration through sports industries and tourism. However, these studies adopt a relatively narrow perspective, overlooking the long-term roles of sports towns in social and cultural development, health improvement, and community building. The multidimensional contributions of sports towns to the poverty alleviation process are not fully explored. Therefore, it is of significant practical importance to conduct an in-depth analysis of the comprehensive roles that sports towns play in targeted poverty alleviation.

Most current research emphasizes the economic benefits of sports towns, particularly their short-term effects in stimulating regional economies through large-scale sports events and tourism. While these studies provide an important foundation for understanding the economic functions of sports towns, they are limited to focusing on the economic boost during the initial development phase, neglecting the potential value of long-term sustainable development and community integration. In contrast, this research aims to explore the broader roles of sports towns in promoting social and cultural development, improving public health, and enhancing overall quality of life for residents, thereby filling this gap in the existing research.

Additionally, the literature rarely systematically explores the deep integration of sports towns with the national targeted poverty alleviation strategy. Although the sports industry can bring economic benefits to impoverished areas, its specific role in building long-term mechanisms for poverty alleviation has not been thoroughly studied. Most research is limited to describing how sports towns promote tourism revenue while neglecting how sports poverty alleviation projects can create long-term employment and social integration for impoverished populations through policy and market mechanisms. This study will analyze the sustainability of sports towns in poverty alleviation, exploring how stable industrial chains can be formed with policy support to achieve long-term sustainable development for local economies.

The existing research also commonly reflects the issue of homogenization in sports towns. Due to excessive imitation of successful models in different regions, many sports towns lack uniqueness and core competitiveness. Although some studies mention this problem, there is insufficient theoretical analysis and practical solutions. To address this issue, this study will conduct an in-depth analysis of typical cases and propose how to combine local resources and cultural characteristics to create uniquely positioned sports towns, achieving differentiation and long-term development.

In conclusion, current research remains weak in both theoretical and practical aspects, especially in areas such as the social benefits, sustainability, and long-term poverty alleviation mechanisms of sports towns. This study will address these deficiencies by proposing innovative solutions and providing empirical analysis to support the sustainable development of sports towns and targeted poverty alleviation in China, fully demonstrating the value and necessity of this research.

## 3 Methods

This study employs two primary research methods: documentary analysis and empirical analysis, both of which are crucial in understanding the role of sports poverty alleviation and model innovation in the development of Chinese sports towns.

### 3.1 Documentary analysis

The documentary analysis method involves systematically reviewing and analyzing various documents and literature relevant to the research topic. This method is used to understand the existing policy framework, historical evolution, and current status of sports towns and their relationship with poverty alleviation.

In this study, key government reports, policies, and academic literature were analyzed. The research focused on official documents such as the Notice on Promoting the Construction of Sports and Leisure Characteristic Towns and Implementation Opinions on the Sports Poverty Alleviation Project. By examining these documents, the research explored the policy context for sports town development and how sports can serve as a tool for rural revitalization.

The specific steps of documentary analysis include:

(1) Collection and Review: Relevant documents were collected and systematically reviewed, including government policies, academic articles, and case studies on sports poverty alleviation and sports town development.
(2) Identification of Key Concepts: From the documents, key themes such as the economic, social, and health impacts of sports towns were identified and used to guide the analysis.
(3) Synthesis: The analysis synthesized information from various sources to provide a coherent framework for understanding the potential of sports towns in driving poverty alleviation.

Through documentary analysis, the research built a theoretical foundation and highlighted gaps in current research, providing a context for further empirical investigation.

### 3.2 Empirical analysis

Empirical analysis was conducted to examine real-world examples of sports town development and its impact on poverty alleviation. This method focuses on collecting and analyzing quantitative and qualitative data from case studies and field observations.

A key part of the empirical analysis involved studying specific sports towns, such as the Mashan Sports Town in Guangxi, a successful model integrating sports with poverty alleviation. By analyzing data on tourism growth, employment, and poverty reduction in Mashan, the research demonstrated how sports towns can create economic opportunities in impoverished regions.

Additionally, interviews and observations provided insights into the local experiences of implementing sports town projects. These qualitative data helped contextualize the quantitative findings, illustrating how sports towns can contribute to both economic development and social cohesion.

In conclusion, the combination of documentary and empirical analysis provided a comprehensive understanding of the challenges and opportunities in developing sports towns for poverty alleviation in China, offering evidence-based recommendations for future model innovation.

## 4 The introduction of the problem

### 4.1 The connotation of sports poverty alleviation

#### 4.1.1 From the perspective of civil rights

From the perspective of civil rights, sports rights are individuals’ rights to engage in or participate in sports, which is a basic human right. Sports poverty alleviation aims to ensure the realization of sports rights among people in poverty-stricken areas. As the subject of this right, the state implements specific sports policies to enable the realization of sports rights for people in sports-poverty areas, fulfills its obligation to protect citizens’ sports rights, and thus ensures the fair development of sports undertakings across various regions and groups.

#### 4.1.2 From the perspective of rural revitalization

The construction of sports characteristic towns provides a solution to the predicaments in the development of a rural revitalization strategy. The comprehensive implementation of the rural revitalization strategy provides policy guarantees for the construction of characteristic sports and leisure towns (16). During the process of promoting economic transformation and upgrading and accelerating the process of rural revitalization, the implementation of sports characteristic towns and the rural revitalization strategy have a high degree of consistency and overlap (1). Firstly, the purpose of implementing the rural revitalization strategy is to promote the transformation and upgrading of rural economy, improve the quality of life of farmers, and accelerate the realization of rural revitalization. Characteristic sports towns are one of the specific industrial forms under the rural revitalization strategy. Second, the development of sports towns can drive the development of related industries, such as tourism, catering, and accommodation in the local area, thereby promoting the industrial revitalization of the countryside and increasing the economic income of farmers (17).

#### 4.1.3 From the perspective of poverty alleviation

From the perspective of the national poverty alleviation strategy, sports poverty alleviation is not implemented as a standalone poverty alleviation policy, but serves a complementary role to economic, cultural, and health poverty alleviation efforts. First, it serves as an effective means of economic poverty alleviation, enhancing regional economic benefits through initiatives such as event-based poverty alleviation and creation of sports complexes. Second, it is intrinsically consistent with cultural poverty alleviation, improving the quality of public cultural services through sports infrastructure, enriching the cultural lives of the impoverished, and guiding cultural concepts with a sports spirit. Lastly, it is a critical link in health poverty alleviation, promoting national fitness through the development of sports and enhancing the framework of health poverty alleviation.

### 4.2 The functions and values of sports characteristic towns in sports poverty alleviation

Against the backdrop of China's decisive stage in poverty alleviation, the practical implementation of poverty alleviation policies to benefit the people and the need for tailored and precise poverty alleviation strategies are issues that must be addressed. Since 2016, when the state proposed the construction of characteristic towns, poverty-stricken areas have utilized their superior natural resources to build sports characteristic towns, providing a platform for targeted and relocated poverty alleviation. As a new model for sports poverty alleviation, sports characteristic towns have unique values and advantages in the battle against poverty.

#### 4.2.1 Economic value

The immediate economic value of sports characteristic towns lies in their ability to create economic benefits for poverty-stricken areas and drive economic development in these regions. First, the guiding mechanism for the construction of sports characteristic towns was optimized by combining the current economic benefits of the sports industry with the long-term sustainable development goals of poverty-stricken areas. Second, the introduction of various enterprises has formed industrial clusters, transforming and upgrading the single agricultural industrial structure of poverty-stricken areas, innovating productivity, and establishing characteristic sports and leisure towns as a series of interconnected and interlocking projects (18). For example, the construction of the Zhangjiakou Chongli Taizicheng Ice and Snow Town after the successful hosting of the 2022 Winter Olympics has integrated and utilized high-level venues and transportation infrastructure, forming an ice and snow tourism resort with a distinctive industrial feature of ice and snow sports equipment production, ice and snow sports product development, ice and snow sports training, and ice and snow culture dissemination. This has deepened the resource sharing mechanism between local poverty-stricken groups and developers, promoted regional economic development, and helped the disadvantaged escape poverty and become prosperous.

#### 4.2.2 Social value

Characteristic sports towns provide employment opportunities for rural populations, enhancing their economic integration. By attracting migrant workers to return and start businesses, linking village elites with poor households for mutual development, and leveraging industry associations or professional talent for on-site guidance, an efficient transfer of human capital occurs between cities and regions. The construction of sports characteristic towns brings advanced technologies, cutting-edge information, and knowledge concepts to impoverished areas, which helps update outdated and backward thinking, promote self-development among the public, and prevent the recurrence of poverty. This breaks the cycle of “getting out of poverty—falling back into poverty—needing to be rescued again”.

#### 4.2.3 Brand value

Sports characteristic towns must not only achieve sustained economic growth, but also fulfill the aspirations of impoverished communities for a better life. By creating differentiated brands, they can showcase the region's unique characteristics. On the one hand, they improve their living infrastructure by building bridges, roads, and water and electricity systems, and increase the construction of industrialized social support facilities to help impoverished communities improve their production and living conditions. On the other hand, by optimizing and integrating the resources of impoverished areas, they transform villages into open, sustainable, and high-level circular economies, promoting harmonious development between people and society, and between people and nature. Furthermore, relying on the unique natural and cultural resources of the local area, characteristic sports towns form sustainable sports industry complexes, which can promote impoverished areas as regional landmarks and attract a wide range of sports enthusiasts, health and wellness seekers, and tourists at different levels.

## 5 The current status and issues of sports characteristic towns in China

### 5.1 The current development status of sports characteristic towns in China

As a representative project, sports characteristic towns in China have benefited from the guidance and support of national policies, leading to a continuous increase in their number and a diverse industrialized development trend. Currently, the construction of sports towns in China is primarily guided by the government. With the promotion of experiences from key national sports town construction projects, various sports towns are emerging across the country. The establishment of sports industry complexes through models such as “Sports + Tourism,Sports + Culture,Sports + Health and Wellness”, and “Sports + Education and Training” have become the main trend in the integration of industrial development (Table 1).

**Table 1:** An overview of national policies related to the construction of sports towns in China.

Time	Issuing authority	Name
2016. 7	The Ministry of Housing and Urban-Rural Development, the National Development and Reform Commission, and the Ministry of Finance	Notice on Initiating Special Town Cultivation Work
2016. 10	General Office of the State Council The National Development and Reform Commission	Guidelines on Accelerating the Development of the Fitness and Leisure Industry Guidelines on Accelerating the Construction of Beautiful and Characteristic Small (Urban) Towns
2017. 5	The General Office of the General Administration of Sport of China	Notice on Promoting the Construction of Sports and Leisure Themed Towns
2018. 3	General Office of the State Council	Guidelines on Promoting the Development of Integrated Tourism
2018. 8	The National Development and Reform Commission	Notice on Establishing Mechanisms for High-Quality Development of Distinctive Small Towns and Small Towns
2019. 3	State Physical Culture Administration	Guidelines for the Construction of Pilot Projects of Sports and Leisure Themed Towns
2019. 9	General Office of the State Council	The Outline for Building a Strong Sports Country The Opinions on Promoting National Fitness and Sports Consumption and Promoting the High-quality Development of the Sports Industry

From the perspective of sports town types, they are categorized into four major types: event-oriented, industry-oriented, leisure-oriented, and health-oriented. They are usually laid out based on the regional geographical environment, integrating multiple elements, such as ecology and culture, and promoting regional development according to local conditions. Among them, outdoor sports are the main focus, with ball sports, ice and snow sports, water sports, car and motorcycle sports, marathons, cycling, and martial arts as the key development types (Table 2).

**Table 2:** The basic types of sports towns in China.

Type	Ancillary services	Typical town
Competition-oriented	Sports towns formed around a major individual sports event as the core, with services related to the event as an extension, and leisure experience activities as a supplement	Zhejiang Haining Marathon Town Beijing Fengtai Football Town Guangxi Mashan Climbing Town
Leisure-oriented	Based on the ecological environment, characterized by the gathering of diverse and highly participatory and experiential sports and leisure activities, formed for mass consumption sports towns	Zhejiang Shaoxing Keqiao Cool Play Town Guangdong Baihehu Sports and Health Characteristic Town Guangdong Zhongshan International Baseball Town
Wellness-oriented	Based on the ecological environment, using sports as the carrier, with health preservation as the main goal, and integrating tourism, resort development to form a health and wellness resort-type characteristic town	Zhejiang Pinghu Jiulongshan Aviation Sports Town Henan Dengfeng Songhuang Sports Town Jiangsu Tangshan Sports Leisure Resort Town
Business-oriented	Based on the production and manufacturing of sports equipment or goods, integrating the upstream and downstream industrial chains, ultimately forming an industrial cluster characterized by the integrated development of the secondary and tertiary industries	Zhejiang Deqing Moganshan Naked Heart Sports Town Sichuan Chengdu Jintang Internet + Football Town Anhui Huangshan International Outdoor Sports Base

**Figure 1:**
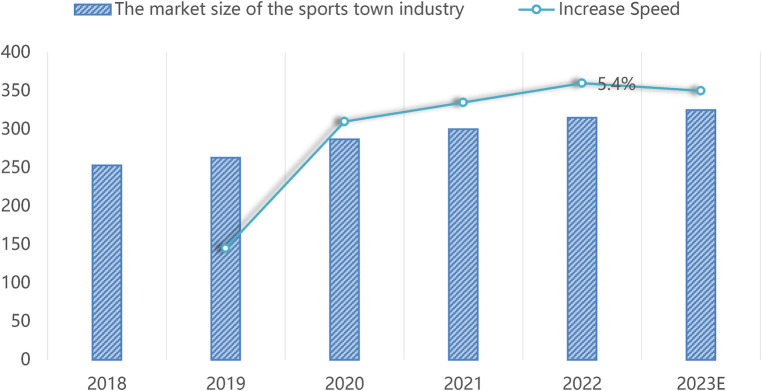
Market size and growth rate of China's sports characteristic town industry from 2018 to 2023 (19).

According to the 2023–2029 market competitiveness analysis and investment prospect forecast report of China's sports town industry released by Market Research Online, by 2022, the market size of China's sports town industry will reach 2.2 trillion yuan, an increase of 23.3% over 2018 and 36.7% over 2017 (Figure 1). The development of the market size of China's sports town industry will be affected by government policies. In recent years, the government has increased its investment in the sports town industry, strongly supported the development of the sports town industry, and the government will continue to strengthen its policy support (19).

### 5.2 The bottlenecks faced by the development model of sports towns in China

#### 5.2.1 Prominent homogenization characteristics

Against the backdrop of China's supply side reform and the integrated development of industries, many investors have focused on sports towns or industrial parks. However, in the planning and construction process, there is often a lack of insight into and utilization of regional characteristics, leading to a trend of blindly following suit, which has caused these developments to deviate from the “sports” track and become predominantly commercial tourism industries. Taking the existing football towns in China as an example, many are invested in and constructed by real estate companies, with their models being largely replicable. Moreover, the tendency towards “heavy asset” development is prominent, and the integration of sports use and commercial nature is not well-defined. Additionally, ice and snow towns are concentrated in the Northeast and the Beijing-Tianjin-Hebei region, which account for 72% of the domestic snowfield resources, and face challenges in achieving diversified operations throughout the year due to issues such as seasonality dependence. Therefore, addressing the problem of homogenization in sports towns across various regions is an urgent issue that must be resolved.

#### 5.2.2 Lack of distinctive core competitiveness

The lack of distinctive core competitiveness is one of the issues affecting the sustainable development of characteristic sports towns. During the planning and construction of some of these towns, issues such as the unprofessional selection of sports content and events, overly ambitious planning, inadequate supporting infrastructure, and absence of core resources have emerged (20). These problems result in the sports characteristics of the towns not being prominently featured and a lack of unique or core content industries to support them. This is particularly evident in the choice of sports events, where many towns blindly followed successful cases without considering the overall context of the town, choosing popular, low-threshold, and simplistic sports events, such as camping, cycling, marathons, and walking. In terms of planning and positioning, there is a tendency to aim for grandeur and simplicity, leading to repetitive construction and unclear themes, positioning, and scopes. In terms of facilities and support services, there is a lack of overall planning centered on sports, with a low degree of industry integration. The content is monotonous and related support service projects are lacking, making it difficult to meet consumer needs.

#### 5.2.3 Insufficient marketization operation

Sports towns are not part of the national administrative system, and differ from ordinary towns and villages. They are more like industrial clusters that integrate sports, culture, tourism, and elderly care, essentially a geographical concept. Integration of industries implies the participation of multiple entities. Combined with poverty alleviation efforts, collaboration among various departments and entities on the same support platform is required. In practice, sports towns often become a means for the government to attract investment and are tied to performance indicators. There is excessive intervention, and the relationship between the government and the market is not yet clearly defined. The government still acts as a manager and lacks healthy interaction with enterprises during the planning and construction processes. Moreover, in the process of poverty alleviation, there is often a focus on short-term increases in residents’ incomes, and the “blood transfusion” approach to poverty alleviation has not yet been transformed.

## 6 Case study: the sports poverty alleviation model of the mashan sports characteristic town

### 6.1 The basis for selecting the case study

Mashan County is located in the middle of Guangxi Province, China, at the northern foot of Daming Mountain and on the south bank of the middle section of Hongshui River. The county seat is 96 kilometers away from Nanning, with a total area of 2,345 square kilometers. It has jurisdiction over 11 towns (including 2 Yao townships) and a population of 570,000. Mashan County is located in a typical karst landform area, with unique rock wall resources, especially the rock wall of Sanjiatun, Guling Town, which has obvious advantages in rock climbing development. In August 2017, China's first rock climbing characteristic sports town has successfully settled in Mashan, and has been selected as the first batch of sports and leisure characteristic town pilot projects in China. The annual number of tourists increased from 2,696,500 in 2016 to 6,038,200 in 2019, with an average annual growth rate of 30.84%; The total tourism consumption increased from 1.707 billion yuan in 2016 to 4.442 billion yuan in 2019, with an average annual growth rate of 37.51%. The contribution rate of the tertiary industry led by tourism to the county's economy is over 60%. In terms of driving poverty alleviation, about 16,500 people were lifted out of poverty through tourism from 2016 to 2019, and the poverty alleviation rate of the poor people reached over 17%. The incidence of poverty in the county also dropped from 20.22% at the beginning of 2016 to 0.84% at the end of 2019.

### 6.2 The “sports + poverty alleviation + cultural tourism + county” poverty alleviation model of mashan

Guangxi Mashan Climbing Town, as one of the first batch of sports town demonstration projects designated by the General Administration of Sport of China, relies on its superior geographical conditions and takes full advantage of ecological resources, such as large stone mountain areas and karst landforms. Combined with local ethnic minority cultural characteristics, it has developed into a climbing town. This has maximized the development potential of the “mountains and rivers’ resources and brought significant benefits to the impoverished local population”.

#### 6.2.1 Event-based poverty alleviation

Mashan County, relying on its rich natural ecological resources such as mountains, caves, and rivers, as well as cultural resources such as Zhuang ethnic minority folk culture festivals, has successively organized a number of sports events. In 2017, the China-ASEAN Mountain Marathon (Mashan Station), themed “Running into the Future in the Mountains”, and the 2018 China-ASEAN Mountain Outdoor Sports and Tourism Conference, with forms of charity runs and sports charity fundraising, achieved the integration of events and poverty alleviation. Moreover, the establishment of the “Camping China” National Youth Outdoor Camping Conference in 2018 and the National Youth Climbing Training Base attracted tourists and athletes through events, creating a regional sports event brand and increasing local residents’ income, establishing a new model of event-based poverty alleviation in Mashan.

#### 6.2.2 Industry-based poverty alleviation

Characteristic sports towns are spatial areas dominated by the sports and leisure industry (4) their core lies in the integrated development of the dominant industry and its related industries. The sports and leisure industry and its related industries rely on supporting platforms to drive the development of sports towns and exert the effects of poverty alleviation and radiation. Mashan County takes outdoor sports such as rock climbing as the dominant industry. While hosting various events, it promotes the development of regional tourism resources, leisure agriculture, and special breeding through measures such as attracting investment and promoting poverty alleviation projects. Simultaneously, the construction of the climbing town directly drives local residents to achieve employment and increase their tourism income. During the 2018 National Day Golden Week holiday alone, Mashan County received 668,400 tourists, with total business revenue of 36.3487 million yuan (21).

#### 6.2.3 Multi-stakeholder participation

According to stakeholder theory, there are many elements in the geographical location of a sports town, and local residents are a key element. Their role is not just as a supplier of labor, but also as a beneficiary of the outcomes. In traditional sports poverty alleviation models, the level of local residents’ participation in regional management is low, often relying on government pairing assistance, and their sports needs have not been met. In the Mashan model, this approach is changed, with the operation and management of the climbing town and outdoor sports park entrusted to local residents, ensuring that economic benefits truly benefit people. Additionally, the construction of sports infrastructure, through sports public welfare fund projects such as the “Snow Carbon Project” and “National Fitness Path”, injects investment funds into sports poverty alleviation in the region, increasing sports public services, developing grassroots mass fitness movements, and gradually breaking down the urban-rural dual structure to achieve co-construction and sharing.

### 6.3 Lessons and warnings

As a poverty alleviation demonstration project in national sports towns, the Mashan model undoubtedly provides a new path for regional poverty alleviation. First, it differentiates its positioning based on regional natural resource endowment. By utilizing unique karst landforms, it selects outdoor sports with the most foundation and growth potential as the core industry and develops them vigorously. Under the advantage of natural resources, other production factors are introduced, forming a regional sports IP with economic productivity and sustainable development potential. Second, people-oriented concepts run throughout. It highlights ethnic minority regional cultural characteristics, based on local history and culture, and at the same time popularizes public sports services. A sports town is not just an economic complex; its residential functions should not be neglected. Third, the government plays a key role in the planning and construction of sports towns. The construction of sports towns cannot be separated from national financial support and policy guidance; however, excessive government intervention may actually achieve the opposite effect. Therefore, the government's role should not be that of a manager or commander but rather that of a guide.

The “Mashan Model” of sports poverty alleviation has provided a new growth pole for regional economic development, while also developing a new model of targeted poverty alleviation. However, some issues have emerged during this process. In 2014, Mashan County was held accountable by the State Council for falsely reporting poverty alleviation targets, with nearly 9% of the recognized poverty alleviation targets being incorrect. Such fraudulent behavior ultimately harms people's interests. In the context of targeted poverty alleviation, it is particularly important to focus on mechanisms for accountability of government behavior and data transparency during the construction of sports characteristic towns.

## 7 The innovative path of the development model of sports characteristic towns under the background of targeted poverty alleviation

### 7.1 Top-level design: incorporating the construction of sports characteristic towns into the national category of targeted poverty alleviation

To give full play to the unique advantages of sports in getting rid of poverty, starting from the planning, design and industrial layout of sports characteristic towns, we should consider how to make farmers become “three-gold farmers” in terms of system and mechanism (farmers can get three incomes: land transfer, asset dividends and employment salary, which are called “three-gold” farmers) (22), and how to make people share the achievements and enjoy happiness. Efforts should be made to explore a new mode of sports poverty alleviation, and the construction of sports characteristic towns should be combined with poverty alleviation.

Specifically, under the background of sports poverty alleviation, the formulation of relevant policies for sports town construction projects in poverty-stricken areas needs to be scientific and targeted. First, in the planning stage of sports towns, attention should be paid to the needs of the local poor people, and industrial design and development should not sacrifice the interests of the masses and the ecological environment. Second, in the construction stage of sports towns, we can give priority to providing employment opportunities to the poor people and organizing skills training, so that local residents can get income and self-development opportunities in the process of accepting poverty alleviation.

Sports town is not composed of a single subject, but a complex with coordinated participation and efficient management of “government-enterprise-residents” multiple subjects. In this complex, local governments, as the key element, provide policy supply and resource support, promote enterprises to play the basic role of industrial town building, drive poor people to realize co-construction and sharing, guide multiple subjects to realize benign interaction, and give full play to the poverty alleviation effect of sports towns with interactive modes such as “enterprise + government + poor people” and “bank + enterprise + government + poor people” (18).

### 7.2 Industry integration: creating a cluster of industries centered on sports and leisure

As an all-round industrial system with intensive elements and spatial agglomeration, sports towns are the general trend of industrial integration and development. The meaning of integration and development is not to pay equal attention to all industries, but to take the leading industries as the core, and then extend the industrial chain and promote the development of related industries. If the characteristic industries of sports characteristic towns want to achieve three-dimensional and multi-dimensional characteristics, outstanding characteristics, extension and sustainability, they need the industrial entities of the towns to continuously extend and develop the industrial chain layout. This requires sports characteristic towns to improve and enrich the industrial chain from four dimensions: value chain, marketing chain, supply and demand chain.

#### 7.2.1 Value chain: deep excavation of the value of sports and leisure products

Online social groups such as “Yuepao Circle”, “Marathon” and “Premier League Live Cheering Group” have opened up a new situation of deep integration and development of Internet, sports and tourism. The trend of leisure and entertainment of public life has obviously enhanced. Mountain sports, water sports, ice and snow sports, golf, camping and watching large-scale competitions have become the hot spots of outdoor sports and leisure. Sports has become a high value-added product for the transformation and upgrading of tourism products, and sports tourism has become a new direction of industrial transformation and upgrading. Through sports products and services, we can meet the upgrade of people's amusement behavior and the spiritual sublimation of self-challenge (23).

#### 7.2.2 Marketing chain: expand the influence of relationship marketing and team marketing

Sports is an important support and platform for the development of human communities and interpersonal communication. Studies have shown that Americans regard “bowling alone” as a sign of the decline of social public life; Germany is the most prosperous country of mass sports clubs in the world, and 1/3 of its citizens join all kinds of sports clubs; There are thousands of amateur football clubs and hundreds of running groups in Beijing; Through the service of the club's marketing department, 60,000 members of Barcelona Club go all over the world to watch games and participate in other sports tourism projects of the club, such as basketball, swimming, tennis, etc. There are more than 1,000 kinds of logo products in a club in Bayern Munich, Germany, which greatly drive the consumption of fans. Sports characteristic towns should focus on sports community marketing and relationship marketing, actively use We Media communication technology for marketing and promotion, save marketing costs, and achieve good and sustained results (24).

#### 7.2.3 Supply and demand chain: differentiated services meet the balance of supply and demand

In the final analysis, the improvement of product innovation ability of sports characteristic towns is the balance of industrial supply and demand. After homogeneous sports town products and dense town layout in the province, it is bound to form fierce competition among customer groups. Apart from towns dominated by characteristic manufacturing, sports towns dominated by sports service can only achieve customer introduction and balance between supply and demand. For example, in October 2016, Jiangsu announced that the first batch of eight sports and health characteristic towns started construction have their own characteristics in content design. Tangshan, Nanjing was originally a “hot spring ten tourism” town, rich in sports resources, slow-moving system and events (25).

#### 7.2.4 Space chain: unicom towns and convenient sharing, forming resource integration

Sports characteristic towns should form the concept of open development and shared development. Actively integrate the surrounding resource endowments and other existing surrounding characteristic town resources, form resource integration ability and drive brands. Actively make use of the theme cultural festivals, theme activities and tourist events of various characteristic towns, and integrate the tourism resources and sports resources of towns near Unicom. In terms of product publicity and promotion, actively link, share services and products together, and jointly design the price sharing and coordination mechanism of products and services (26).

### 7.3 Brand creation: promoting the characteristic development of sports characteristic towns

Facing the bottleneck of “homogenization” mode of existing sports characteristic towns, the creation of characteristic brands is the key to breaking the situation. The key reason why foreign sports towns such as Wimbledon in England and Queenstown in New Zealand can last for hundreds of years lies in the differentiated positioning of their own brands. China's sports characteristic towns should highlight their characteristic development, create characteristic brands and enhance the core competitiveness of the market (1). Characteristic industries. Sports characteristic towns should make use of natural sports resource endowments, integrate the needs and characteristics of sports culture, and gather mature sports events, training, fitness services and leisure activities. It is also necessary to consider the supply and supplement of local public sports facilities, as well as the matching and integration with the extended functions of events, training, tourism, sightseeing, vacation, medical care, education, science and technology, community life, etc (3, 27). Characteristic form. The characteristic form of sports and leisure characteristic towns must be the manifestation form of town characteristics that are unified with characteristic industries and consistent with characteristic functions. The characteristic form can be concentrated in the high integration and unity of production, life and ecological space, the rendering and embellishment of theme features and cultural symbols, and the spiritual commemoration of sports culture and historical inheritance (4). Characteristic mechanism. The characteristic mechanism of sports and leisure characteristic towns is the market development and operation mechanism guided by the government and with enterprises as the main body. The government provides policies on land, finance, talent introduction, evaluation and assessment, incentive measures, public facilities construction, etc., provides the influence and dissemination of government sports events and activities, and provides the ability to integrate resources of various departments. Enterprises gather various production factors and resource endowments, give full play to the advantages of social capital and government cooperation, and increase the supply of sports products and services. The characteristic mechanism provides institutional guarantee for developing characteristic industries, enriching characteristic functions and shaping characteristic forms.

Specifically, the first is to dig deep into national sports resources. At present, China's poverty-stricken areas are mostly concentrated in mountainous areas and ethnic minorities gather, and the original ecological national sports cultural resources are rich, such as Mongolian Nadam and Tujia dragon boat race, which have high sports development value. Second, innovative use of geographical environment advantages, unique natural advantages such as mountains, forests, wetlands and canyons can provide resource guarantee for the construction of sports towns, thus reducing the cost of sports tourism development, and coincide with the original intention of ecological protection.

### 7.4 Self-reliant poverty alleviation: enhancing the self-development capacity of the impoverished population

The construction of sports characteristic towns is an important carrier for coordinating urban and rural development. To gradually form a spatial pattern of coordinated and sustainable development of population, resources and environment, we must adhere to the people-oriented principle, coordinate the layout of production, living and ecological space around people's urbanization, improve urban functions, fill the shortcomings of urban infrastructure, public services and ecological environment, create a livable and industrial environment, improve people's sense of acquisition and happiness, and prevent image projects and real estate development from soaring the cost of production factors such as land.

The construction of sports characteristic towns involves economic restructuring issues such as the change of land nature and ownership, the layout and location selection of clustered industries, the stable employment rate of permanent residents, the change of real estate prices in towns after development, the protection of local residents’ vital interests such as public services, public affairs and public life, and the redistribution of production space and living space. The above-mentioned series of economic reconstruction issues are actually related to social relations and social governance. To make the construction of sports characteristic towns truly enhance people's sense of acquisition and happiness, we must establish a “people-oriented” development concept. Give full play to the important role of people in promoting the deep integration of “production-city humanities”, and promote the coordination and unity of the economic development goals of characteristic industries and the goals of residents’ production, life and self-development.

The key to people-oriented is to provide employment and entrepreneurial opportunities for the poor by developing sports towns, change their inert thinking, improve their self-development ability, and realize hematopoietic poverty alleviation. While developing sports industry, it is also necessary to meet the living and living functions of residents, improve the living conditions in poverty-stricken areas, form a mature residential community, and integrate regional production, life and ecology into it (18). In addition, through infrastructure construction, leisure and health care should be combined with sports to meet the sports needs of local residents, improve people's health level and guide the formation of healthy lifestyles.

## Data Availability

The original contributions presented in the study are included in the article/Supplementary Material, further inquiries can be directed to the corresponding author.
